# Oligometastasis: Expansion of Curative Treatments in the Field of Oncology

**DOI:** 10.3390/medicina59111934

**Published:** 2023-11-01

**Authors:** Ah Reum Lim, Chai Hong Rim

**Affiliations:** 1 Department of Internal Medicine, Division of Oncology, Korea University Ansan Hospital, Korea University, Ansan-si 15355, Republic of Korea; ahreumlim@korea.ac.kr; 2 Department of Radiation Oncology, Korea University Ansan Hospital, Korea University, Ansan-si 15355, Republic of Korea

**Keywords:** oligometastasis, immunotherapy, targeted therapy, radiation therapy, surgery, oligometastatic cancer, local treatment, lung cancer

## Abstract

Oligometastasis is defined as the presence of several limited metastatic lesions and is generally limited to three or fewer than five metastatic lesions. Previously, the treatment of metastatic cancer aimed to alleviate symptoms rather than cure them; however, the use of immunotherapy or targeted therapy has greatly improved patient life expectancy. Additionally, the effectiveness and safety of local treatment have recently been proven for oligometastatic cancers and have significantly improved patient survival and decreased recurrence rates. A few metastatic studies on lung cancer have demonstrated the usefulness of combining radiation therapy and immunotherapy. Recently, local and targeted therapy combinations have shown promising results in treating non-small cell lung cancer, predominantly caused by the epidermal growth factor receptor and anaplastic lymphoma kinase gene mutations, suggesting the potential of these new treatment strategies. It is well known that oligometastasis has better clinical results than polymetastasis; however, research on the biological profile of oligometastasis is still lacking. Studies using circulating tumor DNA and circulating tumor cells are at the initial stages of providing a better understanding of oligometastatic cancers, and the biological characteristics of these cancers may be revealed based on more diverse studies. With the development of these treatments, the prognosis for patients with oligometastatic cancers is steadily improving, and if the biological profile is revealed, customized treatment may be provided.

## 1. Introduction

**Figure 1:**
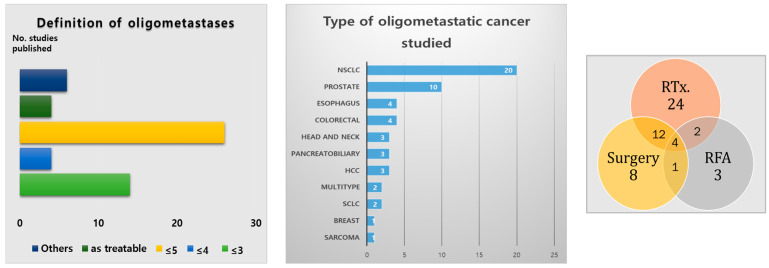
(**Left**) Definition of oligometastasis in studies included in systematic literature analysis, (**Middle**) studies of oligometastasis, (**Right**) treatment methods used as local treatment by study. (Figure adapted from Rim et al. [1]. Role of Local Treatment for Oligometastasis: A Comparability Based Meta-Analysis. Cancer Research and Treatment. 2022. Figures redrawn by authors.)

Oligometastasis is a compound word derived from the Greek word oligo, meaning small number, and metastasis. Although no exact definition has been established, it is generally described as a disease with 3–5 metastatic foci. It is also defined as a disease that can be treated using active local therapies, including surgery or radiation therapy (RT) [1] (Figure 1). Distant metastasis was considered stage 4, and a potential cure was believed to be impossible. However, with the recent development of systemic and local treatment for metastatic disease, the possibility of long-term survival is increasing [2]. Therefore, in this review, we discuss the definition of oligometastasis, recent research trends, and future research directions.

### Overview of Oligometastatic Cancer Treatment

In traditional oncology, cancer spreads throughout the body as tumors invade the primary and surrounding lymph nodes and evolve to a level that invades the blood flow, thereby limiting the potential of local treatment. Local treatments, including surgery or RT for patients with metastatic cancer, provide symptom relief rather than radical treatment. Anticancer treatment can increase the life expectancy of patients with metastatic cancer; however, it is unlikely to cure them, and it can reduce their quality of life.

Oligometastasis is a disease where metastatic lesions are generally limited to fewer than three to five, and local treatment is expected to be beneficial [1]. Since the 1900s, attempts have been made to improve patient quality of life and prognosis by removing metastatic lesions using local treatment. One of the most well-known attempts was the surgical resection of liver metastases from colon cancer. Hughes et al. collected data from 859 patients from multiple institutions (1948–1985) and reported their prognosis after liver metastatic resection when treating colon cancer. They noted a 5-year survival rate of 24.5% and concluded that “survival of >5 years after liver metastatic resection is not only possible but in fact common” [3]. A further study recruited 1568 patients from 85 institutions and reported a 5-year survival rate of 28% [4]. A more recent study (1990–2004), which recruited patients from three institutions in the United States and Europe, reported favorable results (median and 5-year survival rates of 74.3 months and 58%, respectively) [5]. Excellent results have been reported when using local treatment for lung and liver metastases. According to 18 U.S.–European database-centered studies [6], the crude survival rates reached 36%, 26%, and 22% at 5, 10, and 15 years after complete resection of lung metastases of various carcinomas, respectively. Therefore, the authors described lung metastatic resection as a procedure capable of curing lung metastases (Table 1).

In 1995, Samuel Hellman and Ralph Weichselbaum wrote an editorial in the *Journal of Clinical Oncology*, establishing the term “oligometastasis” and proposing it as “a spectrum of disease from purely localized to widespread”. This is clinically significant, as the oligometastatic state could currently be viewed as a point at which to apply the curative strategy, even though it relates to metastatic cancer [7]. This was a novel concept that differed from the step-by-step spreading pattern suggested by Halsted, which is classically accepted as cancer progression, whereby cancer advances step-by-step from the local site to distant metastasis through the lymph nodes and even small metastatic cancer may suggest systemic disease as distant metastasis.

## 2. RT Applications

We hope patients with metastatic cancer will experience a near-cured prognosis (even if only some). However, even after successfully resecting metastatic cancer, approximately two thirds of the patients will experience disease recurrence and metastasis. In this situation, surgical resection can be somewhat burdensome, and depending on the patient’s health status or the location of the metastatic lesion, surgery is frequently difficult. Owing to the development of technology based on computerized tomography, RT can precisely focus high-dose radiation on target tumors and show a local control rate similar to that of early lung cancer surgery [8]. RT is less affected by tumor location than surgery. Therefore, its application as a local treatment for oligometastatic cancers has increased rapidly since the early 2000s.

**Figure 2:**
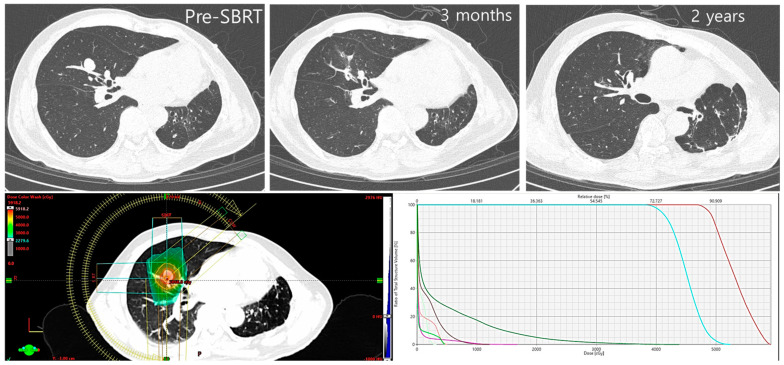
A case of long-term survival obtained by combining local and systemic treatment for oligometastasis. A 60-year-old male patient with multiple small HCC had stable disease for 2 years after trans-arterial chemoembolization. A metastatic nodule (oligorecurrence) was discovered in lung RML, and 55 Gy/5 F SBRT was performed. Remission was achieved at 3 months, and complete remission was achieved at 2 years. Regorafenib was maintained for another 5 years, after performing SBRT, and stable disease was maintained. (**upper**) Sequential image after SBRT; (**lower**) treatment planning of SBRT. The right lower figure shows dose-volume distribution of, from the right, tumor (red), tumor with margin (cyan), right lung (green), heart (magenta), and esophagus (pink). The large gap between dose graphs of tumor target and normal organs such as lung or heart, denotes potent treatment efficacy with small possibility of toxicity. SBRT, stereotactic body radiotherapy; HCC, hepatocellular carcinomas.

Previous studies involving RT for oligometastasis were mainly single-group studies. The results of these studies suggested that, even in cases of metastatic cancer, active RT yielded better results than what is conventionally considered reasonable. Notably, stereotactic body radiotherapy (SBRT), a technique that delivers high doses of radiation to a small target in a short period, has been adopted in treating oligometastatic disease (Figure 2). SBRT has achieved local control similar to those of surgery and embolization, which are existing standard local modalities in treating early lung cancer or intrahepatic malignancies [9,10]. Milano et al. reported a 4-year survival rate of 59% after performing radical-purpose SBRT in 40 patients with breast cancer involving fewer than five metastatic lesions [11]. Kang et al. performed SBRT on patients with colorectal cancer comprising 1–4 metastatic lesions and obtained a 5-year survival rate of 29%, which was similar to the survival period observed in patients after surgical resection of colorectal metastatic cancer [12]. Recently, Chalkidou et al. reported the results of a large-scale study (1422 patients) where SBRT was performed in patients with oligometastatic cancers at 17 cancer centers in the UK. In this study, the 2-year survival rate was 79%, and prostate cancer had a good prognosis (2-year survival rate: 94.6%), while other cancers, including colon cancer (80.3%), kidney cancer (82.4%), breast cancer (83.2%), lung cancer (65.4%), and melanoma (60.5%), had relatively low survival rates [2]. However, overall, the performance of SBRT treatment exceeded the past expectations deemed reasonable for patients with metastatic cancer.

Due to the excellent results obtained from these single-group studies, a significant number of randomized studies were conducted in the mid-2010s. Research was conducted on various carcinomas; however, the most successful areas entailing randomized research were those pertaining to lung and prostate cancers. Gomez et al. [13,14] conducted a randomized study of 49 patients with lung cancer involving no more than three metastases. Patients receiving additional SBRT or surgery showed better results than those receiving standard maintenance alone (median Progression-Free Survival (PFS): 14.4 vs. 7.2 months, *p* = 0.022; and median OS: 41.2 vs. 18.9 months, *p* = 0.017). Furthermore, Iyengar et al. [15] conducted a randomized study comprising 29 patients with lung cancer involving six or fewer metastatic lesions. Patients receiving SBRT treatment reported a better PFS than those solely receiving anticancer treatment (median: 9.7 vs. 3.5 months, *p* = 0.01). These two studies included a relatively small number of patients whose enrollment was prematurely discontinued because of the apparent good progress reported in the group that received local treatment during enrollment. Palma et al. conducted a randomized study of 99 patients with five or fewer metastatic lesions, including patients with lung cancer. The group that underwent SBRT showed better results than the group that received the palliative standard of care (medium survival period: 50 vs. 28 months, *p* = 0.006; median PFS: 11.6 vs. 5.4 months, *p* = 0.001); however, in a randomized study of 86 patients with small cell lung cancer comprising fewer than four lesions in the extended stage, the addition of consolidative extracranial irradiation (thorax + metastatic sites) to Prophylactic Cranial Irradiation (PCI) after chemotherapy did not significantly affect the patient survival rate (1-year survival rate: 50.8% in the RT group; PCI 60.1% in the chemotherapy group; *p* = 0.21). However, the PFS was longer in the consolidative RT group, suggesting a benefit to including the additional radiation treatment (three-month recurrence rate: 14.5% vs. 53.5%, *p* = 0.01) [16] (Table 2).

In addition to lung cancer studies, a randomized study was conducted on the treatment of oligometastatic prostate cancer. Parker et al. [17] conducted research at 117 multicenter institutions in the UK and Switzerland (STAMPEDE trial). They evaluated the utility of prostate RT in addition to male hormone deprivation for metastatic prostate cancer treatment. The addition of RT was beneficial regarding the OS (3-year survival rate: 81% vs. 73%, *p* = 0.007) and failure-free survival (3-year survival rate: 50% vs. 33%, *p* = 0.033), especially in the subgroup with the low metastatic burden (less than three metastatic lesions). In a phase 2 randomized study by Ost et al. [18], androgen-deprivation-therapy-free survival was high in patients with prostate cancer with three or fewer metastatic lesions who underwent metastasis-directed treatment (surgery or RT; median 21 vs. 13 months).

## 3. Current Status of Oligometastasis Studies

### 3.1. Results of Literature Analysis on Oligometastatic Treatment

Over the last decade, many institutions have published research results on the treatment of oligometastasis (including the randomized studies mentioned in Section 2). Recently, radiologists from multiple domestic institutions in charge of treating oligometastatic cancers launched the Oligometastasis Working Group (OWG; Korean Cancer Association) to conduct systematic literature analyses and surveys. The group conducted these analyses on studies reporting on oligometastatic oncological treatment results published until March 2022, and included a control group for local treatment (e.g., anticancer treatment and standard conventional treatment) [1]. Single-group studies were excluded. Of the 54 studies included, 22 (40.7%) and 10 (18.5%) focused on lung and prostate cancers, respectively. Radiotherapy (42 of the 54 studies, 77.8%) was the most common method used as a local treatment, followed by surgical resection (25 studies, 46.3%) and radiofrequency ablation (10 studies, 18.5%). Regarding the definition of oligometastasis, the most common studies defined the number of metastatic lesions as five or fewer (24 of the 54 studies, 48.1%), followed by three or fewer (14 studies, 25.9%). In the OWG meta-analysis, in studies involving five or fewer metastatic lesions, the use of local treatment was beneficial for both the OS (odds ratio: 2.896, *p* < 0.001) and PFS (odds ratio: 3.045, *p* < 0.001). In studies with three or fewer metastatic lesions, the use of local treatment was beneficial for both the survival (odds ratio: 1.535, *p* = 0.016) and the PFS (odds ratio: 1.668, *p* = 0.003) rates. Regarding the survival and PFS in the OWG subgroup analysis that examined non-small cell cancer studies exclusively, the degree of benefit was similar to the values analyzed in this study. However, no local treatment benefit was found in the OWG subgroup analysis of small-cell lung cancer studies.

In the last 2–3 years, studies regarding oligometastatic cancers have focused on additional topics rather than evaluating the benefits of local treatment. With the development of various targeted therapies, studies on local treatment (mainly RT) for oligometastatic cancers, targeted treatment, and immunotherapy are currently being conducted, especially in the field of lung cancer.

### 3.2. Combining Immunotherapy with Radiotherapy in Non-Small Cell Lung Cancer (NSCLC)

The development of immunotherapy has achieved a breakthrough improvement in treating metastatic NSCLC, and advances in RT for NSCLC treatment have been promising with acceptable safety profiles. In the randomized PEMBRO-RT phase 2 study [19], the pembrolizumab maintenance group was compared to the pembrolizumab monotherapy group after SBRT. The PFS was beneficial in the pembrolizumab group after SBRT (median: 6.6 vs. 1.9 months, *p* = 0.19), and the objective response rate at week 12 was significantly beneficial (36 vs. 18%). Notably, in this study, the PFS and overall survival (OS) improved even at 0% of the programmed death-ligand 1 (PD-L1) tumor proportion score (22 vs. 4%, *p* = 0.03). In the randomized MDACC trial [20], one group received RT and pembrolizumab and was compared to the group receiving pembrolizumab exclusively, and no difference was observed in the objective response rate; however, the subgroup analysis showed a significant increase in the disease-free survival of the low-PD-L1-expression group (20.8 vs. 4.6 months, *p* = 0.004), demonstrating the same trend observed in previous studies. Theelen et al. [21] reissued these results by combining the PEMBRO-RT and MDACC studies. The addition of radiotherapy was compared to pembrolizumab treatment alone, and the abscopal effect (treatment response outside the range of RT) and control response rates showed a large difference of 41.7 vs. 19.7% and 65.3 vs. 43.4%, respectively. Additionally, the disease-free survival (median: 9.0 vs. 4.4 months, *p* = 0.0045) and OS (median: 19.2 vs. 8.7 months, *p* = 0.0004) also showed significant benefits when radiotherapy was added. No significant difference was observed in the response rate according to PD-L1 expression; however, at low expression levels, radiotherapy combined with immunotherapy showed a better response rate than pembrolizumab alone. Overall, these studies indicate that the combination of programmed cell death protein (PD-1)/PD-L1 inhibitors and SABR increases the abscopal response, improving the response and survival rates.

### 3.3. Advances in Oligometastatic NSCLC Treatment: Combining Immunotherapy

In oligometastatic NSCLC, Wang et al. [22] conducted a retrospective study on the benefits of combining radiotherapy with PD-1 inhibitor treatment in patients with oligometastatic lung cancer and fewer than four metastatic lesions. In addition to the PFS benefit (median: 13.8 vs. 8.9 months, *p* = 0.035), the abscopal effect reached 41.3%, and a significant synergic effect of immunotherapy and RT was observed. In a retrospective review conducted by Chen et al. [23], 231 patients with synchronous small metastatic NSCLC treated with primary pembrolizumab were analyzed. Among these patients, 76 who received Local Consolidative Therapy (LCT) showed significant improvements in PFS and OS compared to those who did not receive LCT. Bauml et al.’s phase 2 trial included 45 patients with metachronous and synchronous oligometastatic NSCLC who received adjuvant pembrolizumab for 4–12 weeks after prior comprehensive Locally Ablative Therapy (LAT) [24]. They reported a promising PFS of 19.1 months in patients receiving LAT, which represented a significant improvement over the historical outcomes of 6.6 months (95% confidence interval, 9.4–28.7 months; *p* = 0.005) (Table 3).

### 3.4. Advances in Oligometastatic NSCLC Treatment: Combining Targeted Therapy

Studies on combination treatments of local therapy for oligometastasis are currently being conducted in NSCLC, which is primarily caused by specific gene mutations, including the epidermal growth factor receptor (EGFR) or anaplastic lymphoma kinase (ALK) mutations. Wu et al. [25] reported the results of a single-center retrospective study of EGFR-mutant oligometastatic NSCLC during first-line EGFR-Tyrosine Kinase Inhibitor (TKI) treatment. This study included 145 participants who were analyzed in three groups as follows: 51 (35.2%) who received consolidative LAT to all oligometastatic sites (all-LAT group), 55 (37.9%) who received consolidative LAT to either primary tumor or oligometastatic sites (part-LAT group), and 39 (26.9%) who did not receive any consolidative LAT (non-LAT group). This study showed significantly improved PFS and OS in the consolidative LAT (all-LAT) group compared to those in the part-LAT or non-LAT group. The median PFS in all-LAT, part-LAT, and non-LAT groups was 20.6, 15.6, and 13.9 months, respectively (*p* < 0.001), and the median OS was 40.9, 34.1, and 30.8 months, respectively (*p* < 0.001). The results of SINDASTAL, a phase 3 randomized study in China, have been recently published; this study involved 133 patients with oligometastasis (fewer than five lesions) in the first-generation TKI, categorized into TKI and RT (all tumors and metastatic lesions). Benefits for both OS (median: 25 vs. 17.4 months, *p* < 0.001) and PFS (median: 20.2 vs. 12.5 months, *p* < 0.001) were observed in the TKI-RT group [26] (Table 3).

### 3.5. Current Ongoing Studies and Evolving Strategies in Oligometastatic Cancer Management and Classification

The NORTHSTAR, BRIGHTSTAR, and LONESTAR trials are currently being conducted. The NORTHSTAR trial (NCT03410043; a randomized phase 2 study) compares the utility of early vs. optional local treatment in combination with osimertinib treatment, and the BRIGHTSTAR trial (NCT03707938; a single-group phase 2 study) follows the adjuvant brigatinib after 8 weeks of local treatment. After using ipilumamab/nivolumab as an induction, the LONESTAR trial (NCT03391869; a randomized three-phase study) selectively uses local treatment.

Research is also being conducted to further subcategorize the disease status of oligometastatic cancer and perform patient-specific treatment. The European Society for Therapeutic Radiology and Oncology and the European Organization for Research and Treatment of Cancer have classified oligometastatic cancer into nine disease conditions, including de novo oligometastatic disease, repeat oligometastatic disease, and induced oligometastatic disease. This distinction is based on the number of metastatic lesions and the timing of diagnosis [27]. Willman et al. [28] analyzed the data of 385 patients and reported that among the above classifications, induced oligometastatic disease yielded poorer outcomes than de novo or repeated oligometastatic disease (induced vs. de novo median survival: 28.1 vs. 46.3 months, *p* = 0.002; induced vs. repeat median survival: 28.1 vs. 50.3 months, *p* < 0.001). The results of the CURB trial [29], which studied oligoprogression, were recently published by the American Academy of Radiological Oncology. In contrast to the previous major randomized studies on oligometastatic lung cancer, this trial mainly involved de novo oligometastatic disease. Patients with oligoprogression of lung and breast cancer with no more than five metastatic lesions were included in this study. Patients were assigned 1:1 to the SBRT and the conventional palliative care groups. SBRT was beneficial when considering the PFS in the lung cancer group (median: 44 vs. 9 weeks, *p* = 0.004). No benefit of SBRT was observed in the breast cancer group (median: 18 vs. 9 weeks, *p* = 0.5).

## 4. Current Limitations and Prospects

It is well known that oligometastasis has better clinical results than polymetastasis, which includes a large number of lesions; however, studies on the biological profiles of oligometastasis that can demonstrate the state of these independent diseases are still lacking. Lussier et al. [30] reported that miRNAs could distinguish between recurrence- and low-risk groups using samples from patients who underwent surgical resection due to fewer than five lung metastases. Using 17 patient samples, Wong et al. [31] identified three types of miRNAs (miR-23b, miR-449a, and miR-449b) that may predict survival after SBRT for oligometastatic cancer. Hansen et al. [32] suggested that circulating tumor cells (CTCs) can predict prognosis in patients with oligometastasis in the brain resulting from lung cancer; however, only 2 of the 34 patients were positive for CTCs. Referring to recent abstract studies, Lebow et al. [33] investigated ctDNA in patients with NSCLC with 109 oligometastatic diseases and 711 polymetastatic diseases. Significantly lower ctDNA detection rates were observed in the oligometastatic group (48% vs. 67%, *p* < 0.001), and lower detection rates in thoracic metastasis than extrathoracic metastasis (30% vs. 54%, *p* = 0.031). They reported that ctDNA driver mutations influenced the decrease in PFS. Sud et al. [34] considered blood samples before and after definitive RT of patients with ≤5 metastatic sites in ≤3 organs of any malignancy. CTCs were found to decrease from the baseline median value of 28 CTCs/mL to a median value of 15 CTCs/mL on day 30 and a median value of 3.5 CTCs/mL after day 100. Particularly, the group with 15 CTCs/mL or more after 100 days had a significantly higher risk of disorder progression than that with lower values (OR 3.31, *p* = 0.007).

These studies provide an important basis for understanding the biological properties of oligometastasis; however, they are still in their infancy because they are difficult to verify in large-scale clinical studies or for use in actual treatment [35]. Additionally, for cancer cells to evolve into a metastable state, various mechanisms, such as intravasation into the bloodstream, survival in the bloodstream, extravasation, and survival and proliferation at distant sites, are required. In addition to the indicators described above, these various mechanisms are considered to be intricately related to various genetic, molecular, immunological, and clinical factors related to tumor progression [36]. Therefore, future studies should investigate these factors in an integrated manner to identify the possible oligometastatic stages.

## 5. Conclusions

The flow of clinical research on oligometastatic cancer is summarized as follows: Since the beginning of the 20th century, studies have attempted active local treatment for patients with early metastatic cancer to achieve excellent oncological results and open up the possibility of rooting. In the 2010s, these results were established through random- or large-scale studies. In the field of lung cancer treatment, immunotherapy and targeted treatment have recently rapidly developed, and the prognosis has significantly improved for patients with advanced stages of cancer compared to those of the past. These new drugs are thought to be effective in patients with oligometastasis. Thus, future studies should consider the benefit and safety of local treatments, including RT or surgery, and the combined use of these new drugs. Research aimed at enhancing our understanding of the biological properties of oligometastatic cancers is continually advancing, although it may require a considerable amount of time to reach comprehensive conclusions; however, the results will help develop new treatments and provide patient-specific therapies. Various organizations and committees, domestic and abroad, have established clinical definitions and detailed classifications of oligometastatic cancers. Here, the number of lesions, the number and type of organs involved, and the timing of diagnosis are considered. This can improve our understanding of the disease properties of oligometastatic cancers and assist in making therapeutic decisions in the field. Additionally, the active local treatment of oligometastatic diseases may increase the number of radiation treatments, increasing the ecological burden. However, local treatment may have socioeconomic benefits if the cost of managing progressive metastatic disease is reduced and a gain in patient quality of life or survival is obtained [37,38]. Furthermore, it is necessary to study the economic situation and insurance systems of various countries for the active treatment of oligometastasis [39].

## Figures and Tables

**Table 1:** Landmark studies involving liver and lung metastatic resections.

Author, Publication Year	Patient Recruit	No. of Patients	Target Disease	Study Design	Outcomes	Reference
Hughes et al., 1988	1948–1985	697	Colorectal cancer with liver metastasis	Multicenter retrospective	5-year survival: 24.5%	[3]
Nordlinger et al., 1996	1968–1990	1568	Colorectal cancer with liver metastasis	Multicenter retrospective	5-year survival: 28%	[4]
Pawlik et al., 2005	1990–2004	557	Colorectal cancer with liver metastasis	Multicenter retrospective	Median OS: 74.3 months 5-year survival: 58%	[5]
Pastorino et al., 1997	1991–1995	5206	Advanced solid tumor with lung metastasis	Multicenter retrospective	5, 10, and 15-year survival: 36%, 26%, and 22%, respectively	[6]

OS, overall survival.

**Table 2:** RCTs in oligometastatic cancers.

Author, Publication Year	Patient Recruit	No. of Patients	Target Disease	Study Design	Comparison	Outcomes (Months)	Reference
Gomez et al., 2016	2012–2016	49	NSCLC, ≤3 Mets	RCT	RTx. or surgery vs. standard maintenance	PFS 11.9 vs. 3.9	[13]
Gomez et al., 2019	2012–2016	49	NSCLC, ≤3 Mets	RCT	RTx. Or surgery vs. standard maintenance	PFS 14.2 vs. 4.4 OS 41.2 vs. 17.0	[14]
Iyengar et al., 2018	2014–2016	29	NSCLC, ≤6 lesions Including primary, ≤3 Met lung or liver	RCT	SABR + CTx vs. CTx	PFS 9.7 vs. 3.5 OS not reached	[15]
Gore et al., 2017	2010–2015	86	SCLC, extended disease	RCT	PCI and cRT	3-/12-month rate of progression 14.5%/75% vs. 53.3%/79.6%	[16]
Parker et al., 2018	2013–2016	2061	Prostate cancer, newly diagnosed metastatic	RCT	SOC and RTx vs. SOC	failure-free survival 17 vs. 13 no survival advantage	[17]
Ost et al., 2017	2012–2015	62	Prostate cancer, asymptomatic, biochemical recurrence after 1st treatment, ≤3 extracranial Met lesion on PET-CT, and serum testosterone levels > 50 ng/mL	RCT	MDT vs. surveillance	ADT-free survival 21 vs. 13	[18]

ADT, Androgen Deprivation Therapy; cRT, Consolidative Radiation Therapy; CTx, chemotherapy; MDT, Metastasis-Directed Therapy; Mets, metastasis; NSCLC, non-small cell lung cancer; OS, overall survival; PCI, Prophylactic Cranial Irradiation; PET-CT, Positron Emission Tomography–Computed Tomography; PFS, Progression-Free Survival; RCT, Randomized Controlled Trial; SCLC, small cell lung cancer; SOC, standard of care.

**Table 3:** Oligometastatic RCT trials (immunotherapy and targeted therapy).

Author, Publication Year	Patient Recruit	No. of Patients	Target Disease	Study Design	Comparison	Outcomes (Months)	Reference
Wang et al., 2021	2018–2020	152	NSCLC, <4 Mets	Single-center retrospective	ICI + RT vs. ICI	PFS 13.8 vs. 8.9	[22]
Chen et al., 2022	2015–2020	231	NSCLC, ≤5 Mets	Single-center retrospective	LCT vs. non-LCT	PFS 13.97 vs. 10.08 OS 30.67 vs. 21.97	[23]
Bauml et al., 2019	2015–2017	51	NSCLC, ≤4 Mets	Single-arm phase 2 trial	LAT followed by pembrolizumab	PFS 19.1 months (significantly greater than the historical median of 6.6 months)	[24]
Xu et al., 2018	2010–2016	145	NSCLC, ≤5 Mets, EGFR mutant	Single-center retrospective	All-LAT vs. part-LAT vs. non-LAT	PFS 20.6, 15.6, and 13.9 OS 40.9, 34.1, and 30.8	[25]
Wang et al., 2023	2016–2019	133	NSCLC, ≤5 Mets, EGFR mutant	RCT	TKI + RT vs. TKI	PFS 20.2 vs. 12.5 OS 25.5 vs. 17.4	[26]

ICI, immune checkpoint inhibitors; LAT, Locally ablative therapy; LCT, Local Consolidative Therapy; Mets, metastasis; NSCLC, non-small cell lung cancer; OS, overall survival; PFS, Progression-Free Survival; RCT, Randomized Controlled Trial; RT, radiation therapy; TKI, Tyrosine Kinase Inhibitor. All-LAT: LAT to all oligometastatic sites; part-LAT: LAT to either primary tumor or oligometastatic sites.

## Data Availability

Data are available within the article.
